# Search continues: Exploring immunoinformatics platforms for designing an effective multiepitope malaria vaccine candidate

**DOI:** 10.5114/bta/204528

**Published:** 2025-06-30

**Authors:** Charles Osuji, Godwin Etuk-Udo

**Affiliations:** 1 Biotechnology Advanced Research Center, Sheda Science and Technology Complex, Abuja, Nigeria; 2 African Center of Excellence for Oilfield Research, University of Port-Harcourt, Nigeria

**Keywords:** epitopes, immunoinformatics, malaria, Plasmodium falciparum, vaccine

## Abstract

**Background:**

The prevailing public health threat posed by malaria, especially in developing countries, remains a serious concern despite the availability of preventive and control measures. While vaccination offers a powerful means of combating malaria, it has not been fully exploited due to previous unsuccessful attempts before the launch of the RTS,S vaccine. A major challenge in malaria vaccine development continues to be the identification of effective targets capable of eliciting robust immunity, given the complexity of the parasites’ life cycle. Leveraging on the breakthrough of the newly approved malaria vaccine, efforts to develop more effective prophylactic solutions continue with renewed determination.

**Materials and methods:**

In this study, a standard structural bioinformatics pipeline was employed to design a multiepitope subunit vaccine against *Plasmodium*, particularly *P. falciparum*. Thirty subunit epitopes were mined from selected variant surface antigens of *P. falciparum* proteins expressed at different stages of its life cycle, based on their vaccine-likeness. These epitopes were conjugated with suitable adjuvants and linkers into a vaccine construct, which was then subjected to stringent downstream analyses.

**Results:**

Out of an initial pool of 133 epitopes, 30 vaccine-fit epitopes were selected, resulting in a final vaccine construct comprising 570 amino acid residues. This included 12 linear B-cells, 11 cytotoxic T-lymphocytes, and 7 helper T-lymphocyte epitopes, all with favorable predicted structural, antigenic, and physicochemical properties. The construct also demonstrated strong global population coverage (95.04%), robust molecular binding, and simulated immune responses.

**Conclusions:**

With the evolving “Omics” technologies through reverse vaccinology, discovering and designing promising vaccine candidates becomes easier without many challenging experimental rigors. This study highlights the potential of immunoinformatics-aided approaches in accelerating effective malaria vaccine development.

## Introduction

Malaria, a mosquito-borne pathogenic disease, remains a challenging global health concern, with millions of cases reported annually – particularly in Sub-Saharan Africa (WHO 2022). The disease is especially worrisome due to persistently high morbidity and mortality rates, notably among children under five years of age (Dasgupta and Ogbuoji 2022). Caused by protozoan parasites of the genus *Plasmodium* and transmitted by *Anopheles* mosquitoes, five species are known to infect humans: *P. malariae, P. falciparum, P. vivax, P. ovale*, and *P. knowlesi*, with *P. falciparum* being the most widespread and severe (Frimpong et al. 2018; Beeson et al. 2019). The life cycle of this parasite is highly intricate, involving multiple developmental stages in different host systems, which enhances its survival and evasion of host immune defenses (Schieck et al. 2017). This complexity is believed to be a major factor contributing to the persistence and severity of malaria infections despite therapeutic progress.

The high prevalence of malaria in tropical regions – especially in Sub-Saharan Africa (WHO 2017) – can be attributed to climatic conditions favorable to mosquito breeding and inadequate environmental health infrastructure. Vulnerability to malaria is increased by age, environmental, hygienic, and immunological factors as most fatalities occur in children as well as among people living in very poor personal and environmental hygienic states (WHO 2019). According to WHO (2022), 241 million cases of malaria were recorded in 2020, compared to 227 million in 2019, with an estimated 69,000 additional deaths in 2020. This alarming trend reflects the limited impact of existing control efforts, particularly in regions accounting for 93% of global malaria deaths (WHO 2022). The continuing gap between intervention and outcome may be partially attributed to the evolving nature of the pathogen (El-Moamly et al. 2023). Increasing tolerance and resistance to many antimalarial drugs, along with poor compliance with control measures in endemic regions, call for synergistic and multifaceted strategies to eradicate the disease effectively. While the search for more potent therapeutic agents is ongoing, the development of long-lasting, highly efficacious prophylaxes – particularly against *P. falciparum –* remains urgent.

The approval of RTS,S, with demonstrated efficacy, safety, and immunogenicity, marks a significant milestone (Syed 2022; Biamba et al. 2024; Asante et al. 2024). Thus, leveraging this success to develop more effective vaccines with improved features is necessary as RTS,S cannot serve as the solitary vaccine for malaria and it is still undergoing postmarketing surveillance. Also, the increasing tolerance of malaria pathogens that has led to the continual emergence and discontinuation of different generations of antimalarial medications (Thillainayagam and Ramaiah 2016) is another cause for concern. These facts underscore the need for the development of more effective malaria vaccines with improved features like multiple targets, broad-range mechanisms of action, and wider population coverage.

Nonetheless, the journey toward developing effective malaria vaccines began decades ago and has faced numerous obstacles (Maharaj et al. 2021; Chutiyami et al. 2024). Many promising candidates were later found ineffective across geographically diverse antigenic profiles or failed in late-stage clinical trials (Sagara et al. 2018; Kurtovic et al. 2019). Additionally, religious, educational, cultural, and political factors in some endemic regions complicate the acceptance and implementation of novel vaccines, including those for malaria and COVID-19 (Nnaji and Ozdal 2023; Oduoye et al. 2024; Okesanya et al. 2024). Ethical dilemmas during field trials, efficacy, and safety concerns, and public skepticism also present significant barriers to vaccine development and deployment (Debnath et al. 2024; Tukwasibwe et al. 2023; Ghazy et al. 2024).

Despite these challenges, recent innovations in malaria vaccine development have achieved meaningful progress. The most notable achievement is the development and approval of the RTS,S/AS01 vaccine, which has shown efficacy in preventing malaria, particularly among African children. R21/Matrix-M, another promising vaccine candidate is on the verge of scaling through, with high hopes of better effectiveness than RTS,S, exhibiting a remarkable efficacy of about 77% in phase 2 clinical trials (Datoo et al. 2024; Aderinto et al. 2024).

Some novel approaches leveraging emerging technologies – such as nanotechnology, mRNA, and viral vector-mediated systems – are currently being explored at various stages of clinical trials to enhance the development of effective malaria vaccines. These include the use of mRNA vaccine technology inspired by the COVID-19 vaccine development platform, now being applied to malaria vaccine formulation (Chavda et al. 2022; Kanoi et al. 2022; Matarazzo et al. 2023; Tsoumani et al. 2023). Fotoran et al. (2023) developed an alternative malaria vaccination strategy utilizing self-amplifying mRNA (samRNA) derived from alphaviruses, lacking viral structural genes. This study tested a samRNA vaccine based on the *P. falciparum* PfRH5 antigen delivered intradermally via cationic lipid encapsulation. Mice vaccinated with PfRH5-encoding RNA replicons generated parasite growth-inhibiting antibodies that recognized the native protein *in vitro*. The study recommends samRNA as a promising strategy for future malaria vaccine development.

Additionally, Pendyala et al. (2023) successfully designed and produced chimeric multivalent cPfCSP-SpyCatcher-mi3 nanoparticles incorporating the T1/junctional region – a potent antibody-neutralizing epitope not included in RTS,S or R21 vaccines. A combination system of self-amplifying and self-assembling protein nanoparticle–RNA vaccines targeting *Plasmodium* macrophage migration inhibitory factor (PMIF) showed protection at both pre-erythrocyte and erythrocyte stages in animal models (Malik et al. 2024). This approach can target multiple stages of the malaria parasite’s life cycle and may offer durable and stable immunity.

Patel et al. (2023) reported that structure-based design (SBD1) immunogens elicited significantly more potent strain-transcending antibody responses against various *P. falciparum* strains than the AMA1 or AMA1-RON2 complex. While apical membrane antigen 1 (AMA1) and rhoptry neck protein 2 (RON2) are established vaccine targets for blocking blood-stage parasite growth, the SBD1 study underscores its remarkable potential for next-generation malaria vaccines. Baculovirus-induced rapid innate immunity, as described by Emran et al. (2018), has shown promise in liver-stage *Plasmodium* defense and may serve as an innate immune booster in malaria vaccination strategies. Similarly, adeno-associated viruses used in a multivector synergistic strategy have significantly boosted immune responses against malaria pathogens (Hasyim et al. 2023).

Furthermore, the EphA2 receptor-mediated antimalarial potential of phytochemicals from *Taraxacum officinale, Tinospora cordifolia, Rosmarinus officinalis*, and *Ocimum basilicum* was explored computationally by Shaikh et al. (2022). These phytochemicals exhibited high binding affinities, suggesting their potential as agents against cerebral malaria by targeting the EphA2 receptor. Fatimawali et al. (2021), Hermanto et al. (2022), and Gholam et al. (2023) independently conducted computer-aided studies on circumsporozoite proteins, plasmepsins, and apigenin from plants, highlighting their antimalarial activity. Identification of dual-target inhibitors of PfDHODH and PfCytbc1 for *P. falciparum* functional chain disruption was also reported by Nandi et al. (2022). These and many more laudable advances to proffer innovative solutions in the offing highlight the use of diverse approaches in the search for effective means of combating malaria. Notably, advancements in computational immunology are revolutionizing modern vaccinology, playing key roles in many of these milestones. Other notable applications of bioinformatics in malaria vaccine research and development include the *in silico* analysis of hybrid compounds against *P. falciparum* (Sharma et al. 2022), molecular modeling studies using three different QSAR methods for antimalarial agents (Mandloi et al. 2018), and *in silico* gene expression analysis for identifying novel genetic insights into *P. falciparum* resistance concern and possible pathway for effective vaccine development by Jeyabaska et al. (2020), among others.

Furthermore, immunoinformatics-based approaches have also been successfully used for vaccine development against diseases such as Chikungunya, Nipah, and Coronaviruses (Anwar 2014; Ali et al. 2015; Singh et al. 2020). Therefore, the main objective of this work is to design a multiepitope subunit construct derived from selected antigenic VSAs that play key roles in immune evasion and/or cyto-adhesion, to combat *Plasmodium* pathogens and support the development of an effective malaria vaccine candidate.

### Role of surface antigens in effective malaria vaccine development

Normal innate immunity to malaria is established through coordinated interactions between antibodies and lymphocyte-mediated responses, which gradually develop after repeated infections from birth into later life stages (Andrade et al. 2022). Effective immunity through vaccination may therefore require immune responses targeting multiple surface-expressed antigens at various stages of the parasite’s life cycle. This is essential, as the pathogenesis of malaria is influenced by several host and parasite factors, including the sequestration of erythrocytes in the microvasculature and the expression of VSAs (Wahlgren et al. 2017).

VSAs such as *P. falciparum* Erythrocyte Membrane Protein 1 (PfEMP1), Surface-associated Interspersed Gene Family (SURFIN), Repetitive Interspersed Family (RIFIN), Sub-Telomeric Variable Open Reading Frame (STEVOR), and Merozoite Surface Proteins (MSP) are antigenically diverse and often undergo clonal variation to evade host immune responses (Chan et al. 2014). Investigating the roles and mechanisms of these surface antigens as immune targets is vital for addressing antigenic diversity in vaccine design and development (Bhalerao et al. 2024). These VSAs, encoded by respective multigene families and expressed on the surface of infected RBCs, enhance *P. falciparum’s* ability to sequester in different organs, contributing to the severe pathophysiology observed in complicated malaria cases (Andrade et al. 2022).

Targeting *Plasmodium* VSAs – particularly those of *P. falciparum* – is hypothesized to provoke immune responses that interfere with infected erythrocyte sequestration. This may inhibit adhesion and disrupt rosette formation, thereby enhancing the opsonization of infected RBCs for phagocytosis and promoting parasite clearance (Chan et al. 2014). By carefully selecting various VSAs as immune targets, this study aims to design a potentially effective multiepitope malaria vaccine. The work leverages advancing immunoinformatics technologies to support the ongoing fight against malaria by developing faster, more affordable, and efficacious prophylactic solutions – particularly against *P. falciparum*.

## Materials and methods

### Target sequence retrieval

Epitopes of PfEMP1, RIFIN, STEVOR, MSP, and SURFIN were carefully selected from 3D7-derived *P. falciparum* erythrocyte membrane proteins, retrieved from the UniProtKB database (https://www.uniprot.org) and expressed in FASTA format, following the methods described by Apweiler et al. (2004).

### Epitopes prediction

The retrieved amino acid sequences were analyzed to predict epitopes for linear B-lymphocytes (LBL), cytotoxic T-lymphocytes (CTL), and helper T-lymphocytes (HTL) using the Immune Epitope Database (IEDB) webservers (https://www.iedb.org). Each selected VSAVSA sequence was entered into IEDB’s standard prediction pipeline to identify epitopes at defined thresholds, as described by Khan et al. (2023). The predicted epitopes were selected based on their antibody-inducing potential, and MHC class I and II binding affinities, considering factors such as peptide sequences, length, and percentile ranking, in line with Reynisson et al. (2020).

### Epitope selection

The predicted epitopes were subjected to multiple screening steps to eliminate vaccine-unfit candidates by evaluating their antigenicity, allergenicity, and toxicity. Antigenicity was assessed using VaxiJen v2.0 (https://www.ddg-pharmfac.net), while allergenicity and toxicity were evaluated using AllerTop v2.0 and ToxinPred (http://crdd.osdd.net), respectively (Dimitrov et al. 2014; Bashir et al. 2021). Standard default thresholds were applied to ensure the selection of only highly suitable epitopes. Selection criteria included strong antigenicity, nonallergenicity, and nontoxicity.

The selected HTL epitopes were further evaluated for their cytokine-inducing potential. This analysis was conducted using computational tools designed to predict the induction of interleukin (IL)-4 (http://webs.iiitd.edu.in/raghava/il4pred/predict.php), IL-10 (http://webs.iiitd.edu.in/raghava/il10pred/), and interferon-gamma (IFN-γ) (https://webs.iiitd.edu.in/raghava/ifnepitope/application.php) as described by Kumar et al. (2021). Cytokines play a critical role in stimulating immune responses, particularly by activating natural killer (NK) cells and macrophages (Kumar et al. 2021). Only HTL epitopes with at least one cytokine-inducing capacity were selected for further consideration.

### Population coverage study

Population coverage prediction studies of the T-lymphocyte epitopes across various geographical and ethnic populations were conducted to estimate their percentage coverage based on human leukocyte antigen (HLA) distribution. The genotypic frequencies of HLA class I and II binding alleles corresponding to the final T-cell epitopes were computationally assessed to evaluate their global applicability across HLA supertypes. The IEDB Population Coverage Prediction Server (http://tools.iedb.org/population/) was used for this analysis, following the method described by Bui et al. (2006).

### Vaccine construct design

The final multiepitope vaccine candidate was constructed with 570 amino acid residues, comprising 30 subunit epitopes: 12 LBL, 11 CTL, and 7 HTL peptides. These were conjugated with appropriate adjuvants and linkers. Human beta-defensin-3 (UniProt ID: Q5U7J2), retrieved from the Protein Data Bank (RCSB.org), was used as an adjuvant to enhance immune responses, following the methodology of Verma et al. (2023). The EAAK linker was used to connect the adjuvant to the PADRE sequence. CTL epitopes were flanked by AAY linkers, while HTL and LBL epitopes were flanked by GPGPG linkers, by the method described by Madanagopal (2023).

### Vaccine properties prediction and structural analysis

Following epitope prediction, screening, and conjugation, the complete vaccine construct underwent immunological, physicochemical, and structural analyses, based on the methods outlined by Bin-Sayed et al. (2020). Antigenicity was assessed using VaxiJen v2.0 (https://www.ddg-pharmfac.net) and ANTIGENpro (https://scratch.proteomics.ics.uci.edu/). Allergenicity predictions were carried out using AllerTop v2.0 and AllergenFP v1.1 (https://ddg-pharmfac.net/AllergenFP/). The physicochemistry of the vaccine construct was characterized with the ExPASy-ProtParam web tool (https://web.expasy.org/protparam/) to determine its structural integrity and stability according to methods described by Mahmud et al. (2021) and Bashir et al. (2023). Key parameters analyzed included the Grand Average of Hydropathicity (GRAVY), solubility, half-life, instability index, aliphatic index, molecular weight, and amino acid composition (Pandey et al. 2018). The secondary structure was predicted using PSIPRED 4.0 (http://bioinf.cs.ucl.ac.uk/psipred/) via the Psi-BLAST algorithm (Figure 2). Tertiary structure prediction and modeling were performed using the Iterative Threading Assembly Refinement (I-TASSER) tools (https://zhanggroup.org/I-TASSER/) in line with Roy et al. (2010). The values of the template modeling (TM) scores, C-score, and root mean square deviation (RMSD) of the top five models generated by the web servers were carefully analyzed to choose the best 3-D structure by the methods of Wilkins et al. (2008). Refinement of the 3-D, structure was carried out with the GalaxyRefine web server (https://galaxy.seoklab.org/cgi-bin/submit.cgi?type=REFINE) which runs on the CASP10 refining method to evaluate structural stability according to the methods of Kumar et al. (2021). The improved structures were downloaded and the chosen model was determined by the overall quality values and assessments following the methods of Yang et al. (2022). The vaccine construct’s structural validations were carried out with a Ramachandran plot and Z-score via the Procheck (https://saves.mbi.ucla.edu/) and ProSA web tools (https://prosa.services.came.sbg.ac.at/prosa.php) as described by Wiederstein & Sippl (2007). Prediction of the immune response profile of the vaccine construct was carried out using the C-IMMSIM v10.1 web server (https://kraken.iac.rm.cnr.it/C-IMMSIM/index.php) according to the methods described by Rapin et al. (2010). With simulation intervals of 30 days in two consecutive injections, a single injection time step of 1 with no lipopolysaccharide (LPS) option was used as described by Kumar et al. (2021). Other stimulation parameters were left at their default settings, with the maximum simulation step value set to 100 as described by Castiglione (2012). ElliPro web tool of the IEDB server (http://tools.iedb.org/ellipro/) was used for discontinuous B-cell prediction of the vaccine construct following the methods of Ponomarenko et al. (2008). The refined 3-D structure of the vaccine construct was submitted to the ElliPro web server for conformational B-cell prediction with a threshold value set at 0.5. To assess molecular interaction, protein–protein docking between toll-like receptor 4 (TLR-4) and the vaccine construct was performed. The receptor was retrieved from the Protein Data Bank and preprocessed using Biovia Discovery Studio 2017 to remove ligands, water molecules, and heteroatoms. Binding site prediction and analysis were carried out using the CASTp server (http://sts.bioe.uic.edu/castp/index.html?1bxw), according to Binkowski (2003). Docking was performed using ClusPro v2.0 (https://cluspro.org/help.php), which operates on the PIPER algorithm, as described by Kozakov et al. (2017). The clustering strength and binding energies of the top-ranked docking models were analyzed (Table 7) to determine the stability and interaction potential of the complex.

## Results

**Figure 1:**
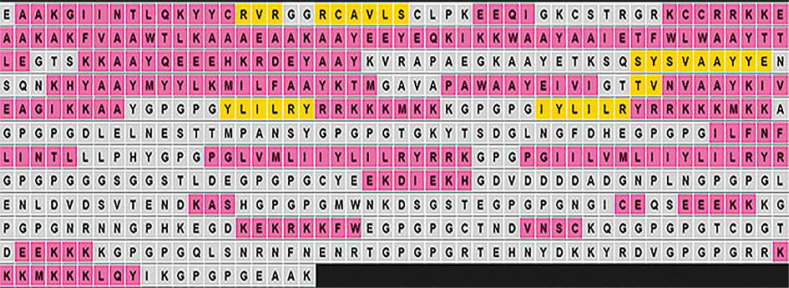
Vaccine candidate sequence

This study employed comprehensive immunoinformatics approaches to identify, conjugate, and evaluate multi-epitope malaria vaccine candidates. The analyses focused primarily on epitopes derived from *P. falciparum* VSA proteins known to play critical roles in malaria pathogenesis. A total of 133 potential epitope sequences were initially predicted from the five selected VSAs, out of which only 30 passed the computational screening and proceeded to the conjugation stage. These final epitopes were linked with appropriate adjuvants and linkers to form the vaccine construct, as illustrated in Figure 1.

**Figure 2:**
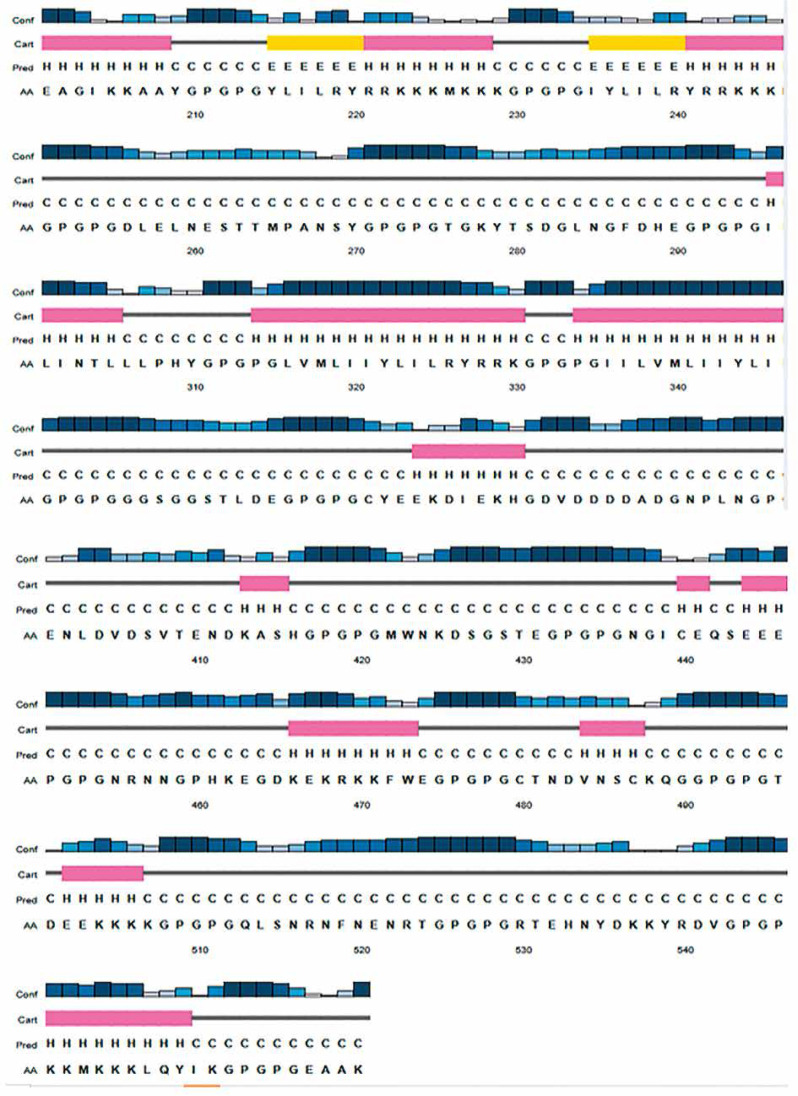
Secondary structure of the vaccine candidate

### Epitopes prediction and screening

Epitope prediction using the IEDB server revealed 69 potential B-cell epitopes across the five VSAs, from which 12 were selected based on antigenicity, allergenicity, and toxicity screening criteria. Similarly, 11 of the 30 predicted CTL epitopes and 14 of the 34 HTLs, were scaled through the antigenicity, allergenicity, and toxicity tests. For cytokine-inducing capacity, only 7 out of the 14 HTLs initially screened for immunological tests scaled through the cytokine-inducing ability tests (Supplementary Tables 1–3).

### Population coverage analysis

The final T-cell epitope population coverage analysis (combined for classes I and II) demonstrated that the selected 18 epitopes provided a global coverage of 95%. For the individual regional coverage, Europe has the highest percentage of 99.96% along with regions like North America, East Asia, North Africa, and West Indies which all have 99% and above (Table 1). Except for Central America which recorded 53% predicted coverage, all other regions had above 94% predicted population coverage. The combined global coverage average was 95.04% consisting of class I and II with 57.97% and 43.03% coverage respectively, and a standard deviation of 10.78.

**Table 1:** Population coverage analysis

S/N	Population/Area	Class combined			Pc90^c^
		Roof^a^ (%)	Average_ hit^b^		
1	World	95.04	25.33	13.37
2	Central Africa	94.79	21.44	9.06
3	Central America	53.8	4.79	1.52
4	East Africa	97.08	23.94	10.89
5	East Asia	99.67	31.79	18.79
6	Europe	99.96	37.22	23.94
7	North Africa	99.01	28.19	16.01
8	North America	99.89	35.17	21.51
9	Northeast Asia	97.88	24.6	11.9
10	Oceania	97.88	23.07	11.84
11	South Africa	95.27	21.54	10.6
12	South America	95.15	20.72	9.76
13	South Asia	98.7	26.29	13.13
14	Southeast Asia	97.66	24.25	11.85
15	Southwest Asia	95.79	22.06	10.68
16	West Africa	98.43	27.15	12.84
	West Indies	99.69	32.98	19.61

Roof^a^ (%) – projected population coverage. Average_ hit^b^ – average number of epitope hits/HLA combinations recognized by the population. Pc90^c^ – Minimum number of epitope hits/HLA combinations recognized by 90% of the population

### Vaccine construct’s physico-chemical and structural analysis

The final vaccine construct comprised 570 amino acid residues, including the human beta-defensin-3 adjuvant, PADRE sequence, and EAAK, AAY, and GPGPG linkers (Figure 1). Strong antigenicity and nonallergenicity of the construct were confirmed using two independent web-based tools (Table 2). Physicochemical analysis indicated the construct had a chemical formula of C2751H4316N778O811S21, a solubility scaled value of 0.851, molecular weight of 61.94 kDa, theoretical isoelectric point (pI) of 9.44, aliphatic index of 58.65, instability index of 36.32, and a Grand Average of Hydropathicity (GRAVY) score of –0.813. Secondary structure prediction using PSIPRED (Figure 2) revealed an α-helix content of 22.7%, β-strand content of 5%, and random coil proportion of 72%.

**Table 2:** Physicochemical and immunological properties of vaccine construct population.

Parameters	Tool	Value	Status
**Immunological**			
Antigenicity	VaxiJen 2.0	1.0251	+
	AntigePro	0.931	+
Allergenicity	AllerTop 2.0	–	–
	AllergenFP	–	–
**Physicochemical**			
Chemical formula	ProtParam	C_2751_H_4316_N_778_O_811_S_21_	Okay
No of amino acids	ProtParam	570	Good
Solubility	Protein-Sol	0.851	Good
Molecular weight	ProtParam	61.941 kD	Good
Theoretical pI	ProtParam	9.44	Good
GRAVY value	ProtParam	–0.813	Hydrophilic
Extinction coefficient	ProtParam	92765	Good
Instability index	ProtParam	36.32	Very stable
Aliphatic index	ProtParam	58.65	Thermostable
Half-life	ProtParam	– 1 h (mammalian reticulocytes) *in vitro* – 0.5 h (Yeast) *in vivo* > 10 h (*Escherichia coli*) *in vitro*	Okay

GRAVY – grand average of hydropathicity, Hu.RBC – human red blood cells, + present, – absent

**Figure 3:**
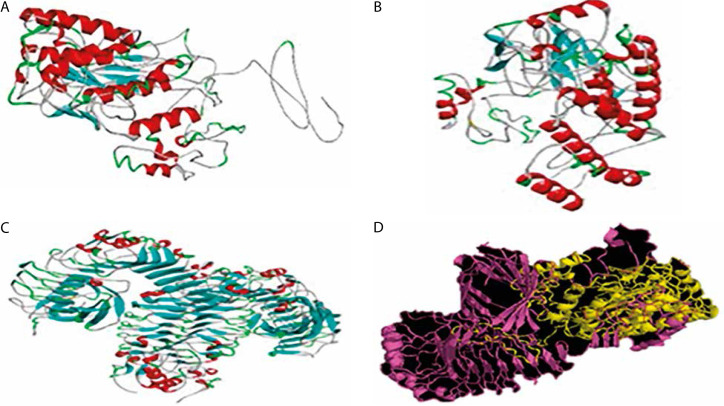
3-D structures of the vaccine candidate at different stages. **A**) 3-D structure of the vaccine candidate. **B**) 3-D modeled structure of the vaccine candidate. **C**) 3-D structure of the TLR-4 receptor. **D**) Docked structure of the vaccine candidate and TLR-4 receptor

Among the five 3D models generated via I-TASSER, model 1 was selected (Figure 3A) and further refined using the GalaxyRefine server. Of the five refined outputs, model 2 (Figure 3B) was chosen based on quality parameters, including a Global Distance Test High Accuracy (GDT-HA) score of 0.8957, RMSD of 0.591, clash score of 12.0, poor rotamer score of 0.4, Ramachandran favored score of 86.1%, and a MolProbity score of 2.231 (Table 3).

**Table 3:** 3-D model structure information

Model	GDT-HA	RMSD	MolProbity	Clash score	Poor rotamers	Roma favoures
Initial	1.0000	0.000	3.181	5.8	17.3	58.7
MODEL 1	0.8761	0.621	2.228	12.1	0.2	86.4
MODEL 2	0.8957	0.591	2.231	12.0	0.4	86.1
MODEL 3	0.8855	0.606	2.223	11.7	0.4	85.9
MODEL 4	0.8829	0.615	2.194	11.1	0.0	86.4
MODEL 5	0.8829	0.617	2.194	11.1	0.7	86.4

**Figure 4:**
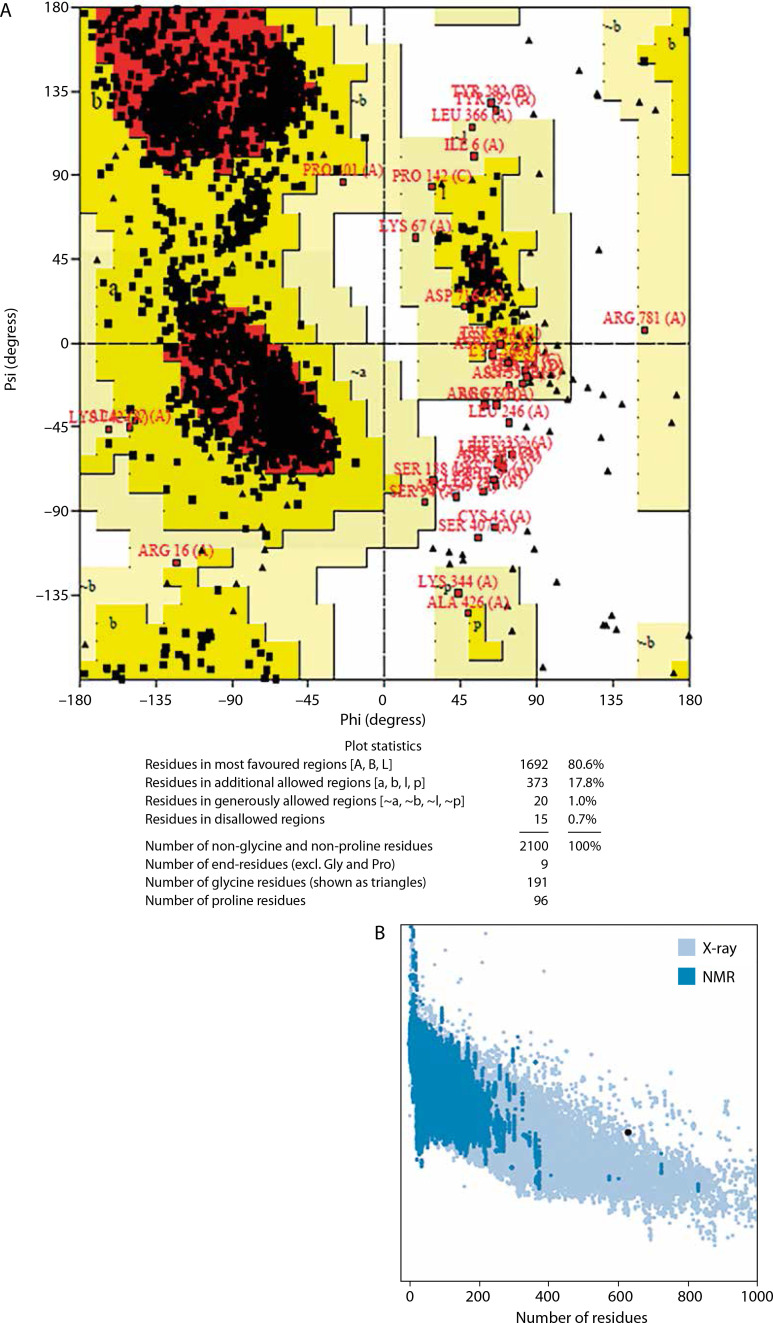
Structural analysis result. **A**) Ramachandran plot. **B**) Z-score

Validation results from PROCHECK and ProSA web servers, displayed via Ramachandran plots and Z-score analyses (Figure 4A–B), indicated that 80.6% of residues were in the most favored regions, 17.8% in additionally allowed regions, 1.0% in generously allowed regions, and only 0.7% in disallowed regions.

### Conformational epitope identification

Based on the standard threshold value of 0.5, eleven discontinuous B-cell epitopes from the vaccine construct containing 308 amino acid residues were identified and selected using the ElliPro server. The sizes of the epitopes were between 5 and 119 residues, with score ranges of 0.508 to 0.931. The individual scores and corresponding 3D representations of the predicted ligand–protein interaction models are presented in Figure 6 and Supplementary Table 4.

**Table 4:** Molecular docking structures information

Cluster	Member	Representative	Weighted score
0	38	Center Lowest energy	–1096.9 –1158.0
1	37	Center Lowest energy	–1056.8 –1184.1
2	33	Center Lowest energy	–1330.2 –1368.6
3	30	Center Lowest energy	–1232.2 –1340.2
4	29	Center Lowest energy	–1050.9 –1167.2
5	29	Center Lowest energy	–1024.3 –1100.7

### Protein–protein docking and immune simulation

**Figure 5:**
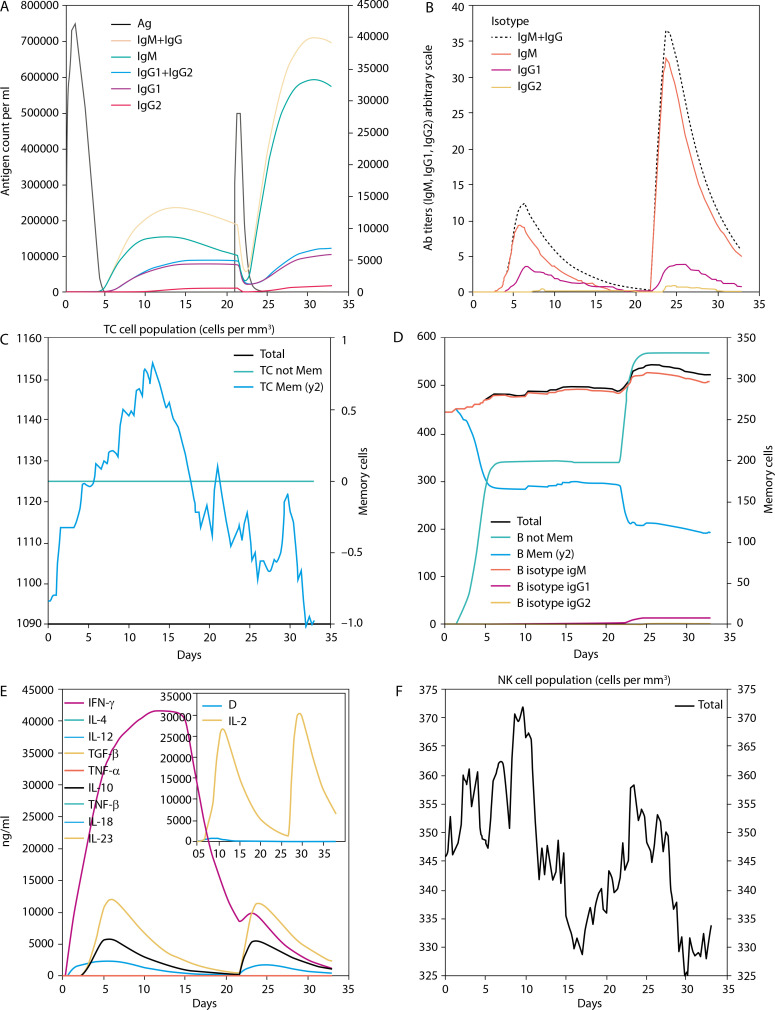
Immune simulation chart result. **A**) Antigen and immunoglobulin. **B**) Plasma B-lymphocyte counts (IgM, IgG1 and IgG2). **C**) CD8 T-cytotoxic lymphocyte counts. **D**) B-lymphocyte total count. **E**) Natural killer cells count. **F**) Cytokines concentration

Molecular docking was performed to evaluate the binding affinity and interaction between the vaccine construct and the TLR4 receptor (Figure 3C). ClusPro 2.0 was used to generate multiple docked complexes, each associated with specific cluster members and energy scores (Table 4). Among these, Cluster 0 – having the highest number of members and the lowest energy score at the center – was selected as the top-ranked docked complex (Figure 3D). Immune response simulation using the C-ImmSim server revealed a characteristic increase in IgM levels following the first vaccine dose. A remarkable increase in gG1, IgG1+IgG2, IgM, and IgG+IgM levels of antibodies after the second dose was also observed (Figure 5).

**Figure 6:**
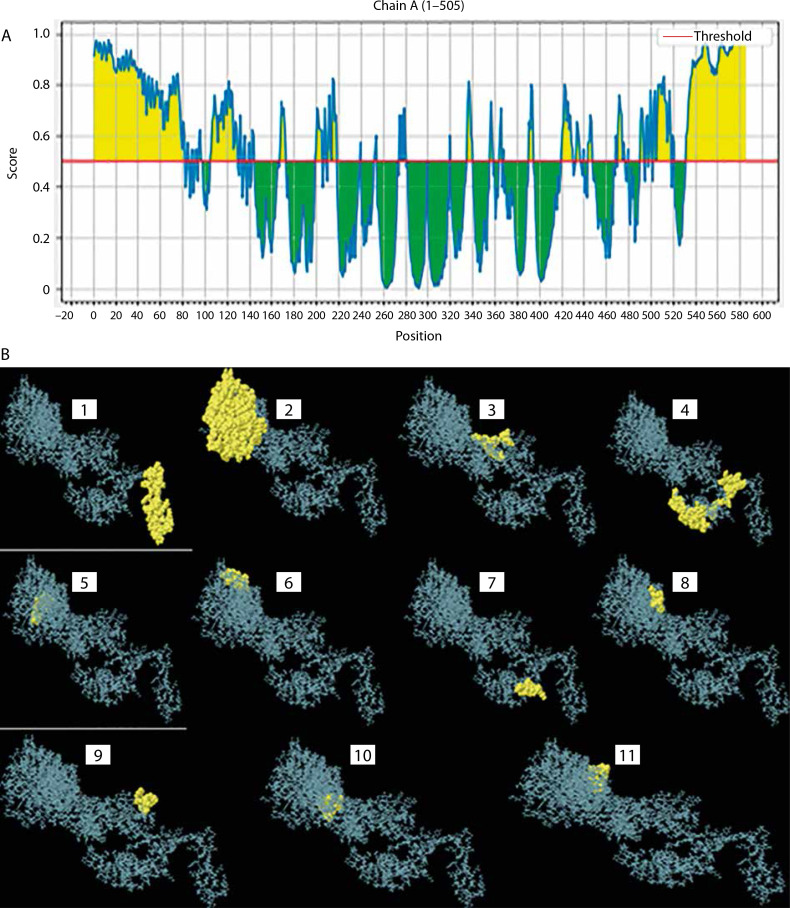
Conformational B-cells identification result. **A**) 2-D score chart of conformational B-cell epitopes. **B**) 3-D representation of conformational B-cell epitopes

## Discussion

*In silico* epitope prediction involves identifying peptide sequences within antigens that are capable of eliciting immune responses, using computational tools. Multiepitope vaccines are thus composed of carefully selected and conjugated subunit epitopes, offering high specificity, enhanced safety, increased stability, and lower production costs (Rostaminia et al. 2023).

In the present vaccine design process, VSAs were selected and screened for vaccine-fit potential using computer-aided prediction and filtering protocols. The first phase involved identifying all probable HTL, CTL, and LBL epitopes, followed by conjugation of the selected subunit epitopes with suitable adjuvants and linkers. This step was essential to stabilize the construct and enhance its immunogenicity.

Subsequent physicochemical, structural, and immunological analyses demonstrated that the final vaccine construct showed good physicochemical properties which are indicative of good stability, solubility, hydrophilicity, thermo stability, and *in vitro* and *in vivo* halflife values which are all in agreement with (Pritam et al. 2019; Hebditch et al. 2017; Ali et al. 2017).

The physicochemical characterization revealed that the vaccine construct possesses an acceptable molecular weight of 61.941 kDa and an instability index of 36.32 – both below the respective upper limits of 110 kDa and 40.0 – consistent with findings by Kumar et al. (2023). These values are indicative of favorable handling stability and ease of purification. The aliphatic index (58.65) and GRAVY score (–0.813) suggest good thermal stability and hydrophilicity, respectively, which are essential for water molecule interactions, as described by Atapour et al. (2020). The solubility and pI values of 0.851 and 9.44, respectively of the construct are within the acceptable ranges. According to Mehlina et al. (2006), the pI is one of the most essential factors when evaluating expression solubility for *P. falciparum* targets. The construct’s pI is balanced – neither too acidic nor too basic – thereby promoting structural stability and immunogenicity, in agreement with Habib et al. (2023).

Estimated half-lives for the construct were 1 h *in vitro* in mammalian reticulocytes, 30 min *in vivo* in yeast, and over 10 h *in vivo* in *Escherichia coli*, indicating suitability for both *in vitro* and *in vivo* preclinical investigations. The secondary structure prediction (Figure 2), with a moderate alpha-helix, low beta-strand, and a high proportion of random coils, suggests structural flexibility—beneficial for antigen functionality, particularly at active binding sites. The percentage of buried, moderately exposed, and exposed residues of the vaccine construct represents good stability and activity in line with Chengxin (2017) and Wei Zheng (2021).

The chosen 3-D structure in PDB format from the I-TASSER server was based on the highest C-value signifying better confidence in the protein model as earlier described by Wei Zhen (2021). Structure refinement and validation followed GalaxyRefine outputs, with evaluation metrics including GDT-HA, clash score, MolProbity, RMSD, poor rotamer score, and Roma favored regions – criteria supported by Hoe et al. (2013) and Yeni and Nining (2022). These metrics were carefully considered to ensure the selection of stable immunogenic structural models of the vaccine candidate which is critical in vaccine research and development. A higher GDT-HA score depicts better residues’ spatial arrangement which is crucial for biological functionality. In comparison, a lower clash score is preferable because it indicates more stability among the individual atoms of the model (Srivastava et al. 2020). The MolProbity gives overall models geometric assessment in terms of torsion and bond angles thus, higher values are preferable as they indicate better geometry. The RMSD is the average distance between the constituent atoms of a model while Poor Rotamer denotes the side-chain conformations and the significant deviation from normal positions. Thus lower values of both RMSD and Poor Rotamer are preferred as they imply more structural stability and closer agreement with the reference model (Olawale et al. 2022). Also, the percentage of residues in the favored region (Rama Favored) is crucial in the functional and structural nature of the construct by Yeni and Nining (2022). The 3-D model structure depicted in Figure 3A, obtained from GalaxyRefine web tool and validated using Ramachandran plot and Z-score values was used to determine the overall structural quality and stability. The values of the Ramachandran plot and Z-score that evaluate the structural validity of the construct showing the region of most allowed and disallowed areas as earlier described by Kumar (2021) were in agreement with Madanagopal (2023). The vaccine construct demonstrated good structural validity with more than 80% atoms in the most favored regions of the Ramachandran plot as shown in Figure 4A. An indication of the good quality of the protein’s backbone torsion angles is in line with Sobolev et al. (2020). The Z-score of the construct (Figure 4B) also corroborates the structural validity of the construct indicating a good fit between the structure and the expected structural features. To ensure global effectiveness, CTL and HTL epitopes were evaluated for population coverage using the IEDB tool. The predicted coverage was 95.04% globally, with an average of 98% in malariaendemic Sub-Saharan African regions, supporting its potential as a promising malaria vaccine candidate (Martinelli 2022; Sanami et al. 2023). Effective immune response stimulation depends on the interaction between the vaccine molecule and host receptors. TLR4 binding activates both innate and adaptive immune responses, initiating a cascade of immunological reactions, as described by Islam et al. (2022). Docking results from ClusPro showed strong binding stability and compactness, as indicated by large cluster sizes and favorable energy values. The choice of the best vaccine–receptor complex was based majorly on cluster size as ClusPro output structures are ranked based on cluster population rather than just energy value by Desta et al. (2020). Thus, the ClusPro structure with the largest cluster members was considered to be of better docking results. Immune simulation results from the C-ImmSim server aligned with typical vaccine-induced responses. The vaccine elicited high IgM levels following the first dose and a marked increase in IgG1, IgG1 IgG2, IgM, and combined IgG+IgM antibody levels after the second dose, suggesting strong B-cell and HTL responses and the potential for long-term immunity (Serwanga et al. 2024). The secondary responses were remarkably higher than primary responses leading to a cascade of other immune-defensive reactions including spiking levels of IgG1+IgG2, IgM, and IgG+IgM antibodies (Vaillant et al. 2023). Additionally, a high presence of HTLs was recorded, reflected by elevated levels of macrophages and NK cells, consistent with the findings of Michel et al. (2013). Conformational B-cell epitopes play critical roles in antigen recognition, as many B-cell targets are conformational in nature (Laver et al. 1990). While this study presents a potentially effective vaccine design based on *in silico* simulation platforms, it acknowledges the extrapolative limitations inherent in such approaches and emphasizes the necessity of *in vitro* and *in vivo* validations.

## Conclusions

The rapid and successful advancement of immunoinformatics-based vaccine development during the COVID-19 pandemic, along with the groundbreaking release of the RTS,S malaria vaccine, has served as a powerful inspiration for renewed efforts in the search for more effective malaria vaccines. Given the immense health burden posed by malaria – particularly in endemic African regions – there is a critical need for more efficacious and broadly protective vaccine candidates. Building upon the significant milestone of the RTS,S vaccine, this work underscores the growing importance of immunoinformatics in advancing multifaceted approaches to malaria vaccine development. Our design of a 570 amino acid residues stable vaccine construct via the application of *in silico* predictive vaccine platforms revealed a significant feat in vaccine development, thereby accelerating the bench-to-bed process in the malaria eradication drive. Through stringent analyses of potential antigenic peptides using standard computer-aided algorithms in correlation with established immunological concepts, this study has designed a stable epitope-guided subunit vaccine candidate with promising efficacy and wide population coverage. Future research is recommended for empirical *in vitro* and *in vivo* validation to authenticate our findings and advance to feasible vaccine development.

## Data Availability

All data generated or analyzed during this study were results of this research work via pipeline tools and webservers and not individual information.
